# Late-onset granular intra-amniotic infection following amniotic membrane transplantation

**DOI:** 10.1016/j.ajoc.2021.101221

**Published:** 2021-10-08

**Authors:** Shaker O. Alreshidi, Samar A. Al-Swailem

**Affiliations:** a Division of Anterior Segment, King Khaled Eye Specialist Hospital, Riyadh, Saudi Arabia; b Division of Anterior Segment, Medical Collage, Majmaah University, Riyadh, Saudi Arabia

**Keywords:** Microbial keratitis, Amniotic membrane, Corneal ulcer, Amniotic membrane transplantation, Therapeutic keratoplasty

## Abstract

**Background:**

The amniotic membrane (AM) is used in ocular surface reconstruction and is effective at promoting epithelialization and preventing corneal perforation in cases of acute microbial keratitis. Here, we report a case of isolated AM infection after AM transplantation for a persistent epithelial defect following tectonic penetrating keratoplasty.

**Case presentation:**

A 47-year-old man with poorly controlled type 2 diabetes mellitus presented to the emergency department with a referral for perforated microbial keratitis. After ophthalmic examination, corneal scraping was performed, and corneal gluing was attempted and failed. Hence, the patient underwent tectonic penetrating keratoplasty. After keratoplasty, the patient developed a persistent epithelial defect that required AM transplantation as an overlay. Thirty days post-AM transplant, the patient presented with signs and symptoms resembling granular microbial infection of the cornea. After two days, the granular findings began dislodging from the corneal surface and were sent for culture, sensitivity, and histopathological identification. Histological analysis of the granular material indicated it to be a small piece of AM stroma infiltrated with mixed-type inflammatory cells. Corneal scraping cultures indicated *Streptococcus mitis* and *Streptococcus oralis*.

**Conclusion:**

The infiltrate was localized to the basement membrane of the AM as, despite the anti-inflammatory effects of AM, it can also act as a barrier against polymorphonuclear leukocyte infiltration from the tear film and microbial invasion into the cornea.

## 1 Introduction

The amniotic membrane (AM) can exert anti-inflammatory and antimicrobial effects, and contains antiangiogenic factors and protease inhibitors. It is effective at promoting epithelial healing and reducing inflammation, scarring, and angiogenesis. The anti-inflammatory effect of AM is likely due to the AM stroma stimulating the apoptosis of inflammatory cells, suppressing cytokines, and containing proteinase inhibitors. In addition, the amniotic membrane acts as a physical barrier against the adhesive surface, potentially preventing infiltration of polymorphonuclear leukocytes from the tear film.^1^^,^^2^

In this report, we present an atypical case of microbial infection 30 days post-AM transplant that mimicked microbial keratitis and was confined to the amniotic membrane. To the best of our knowledge, this is the first report of intra-amniotic infection after amniotic membrane transplantation (AMT).

## 2 Case report

A 47-year-old man with poorly controlled type 2 diabetes mellitus presented to the emergency department in November 2018 with a medical referral for perforated microbial keratitis after undergoing tectonic penetrating keratoplasty (TKPK) at another institution. At presentation, his visual acuity was light perception with poor projection. Corneal scraping was performed for microbial culture and sensitivity, and topical moxifloxacin Q1H was initiated.

One day after admission, ophthalmic examination indicated corneal melting with profuse leakage. Corneal gluing was attempted but failed. Therefore, the patient underwent a tectonic penetrating keratoplasty (TPKP). After keratoplasty, the patient was prescribed topical medication (moxifloxacin QID) and prednisolone acetate 1% QID and systemic oral prophylactic valacyclovir for presumed herpetic infection based on history. Due to the persistent epithelial defect, an amniotic membrane overlay was performed. The membrane was 15 × 15 mm in size, placed with the stromal side down and sutured to the episclera with 9.0 Vicryl sutures using a purse-string technique. A 22-mm bandage contact lens was placed at the end of surgery, and a temporary temporal tarsorrhaphy was performed. The patient was discharged on the same medication regimen in addition to oral doxycycline tablets. The first corneal scraping culture indicated *Streptococcus mitis* and *Streptococcus oralis*. Culture and sensitivity for both the donor corneal rim and AMT remnants were negative.

**Fig. 1:**
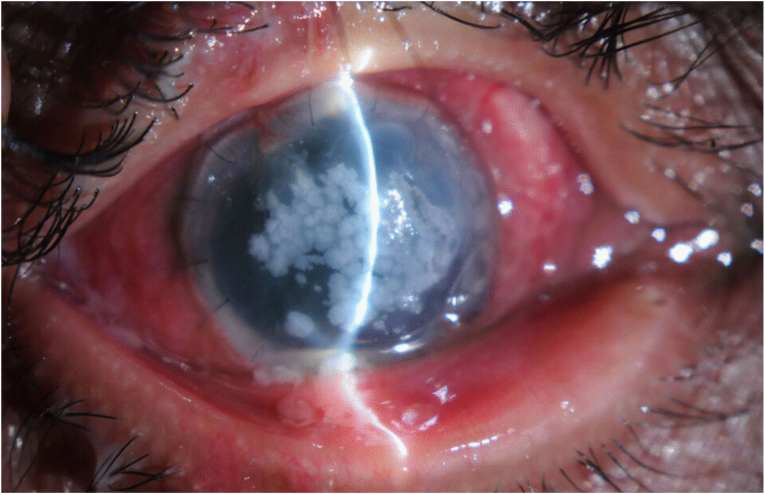
Partially absorbed amniotic membrane with a large whitish granular corneal membrane 40 days after tectonic penetrating keratoplasty.

Forty days following TPKP and 30 days following AMT, the patient continued to use topical moxifloxacin. After releasing the tarsorrhaphy and removing the contact lens, we noted that the AMT was partially absorbed, with a large whitish granular corneal membrane that stained with fluorescein and resembled corneal microbial infection (Fig. 1). The granular appearance and consistency of the amniotic membrane raised suspicion of inflammatory infiltrate versus a secondary intra-amniotic infection. However, the cornea surrounding the granular membrane revealed minimal haze, absence of edema, or infiltration. Due to the negative culture of the amniotic membrane remnant, we decided to maintain the membrane and start topical gentamicin, while retaining prednisolone acetate 1% twice a day, and frequent lubrication. After two days, the granular membrane started to partially dislodge from the corneal surface, and a specimen was sent for culture, sensitivity testing and histopathological identification. Re-epithelialization was poor, and there were signs of persistent epithelial defects.

**Fig. 2:**
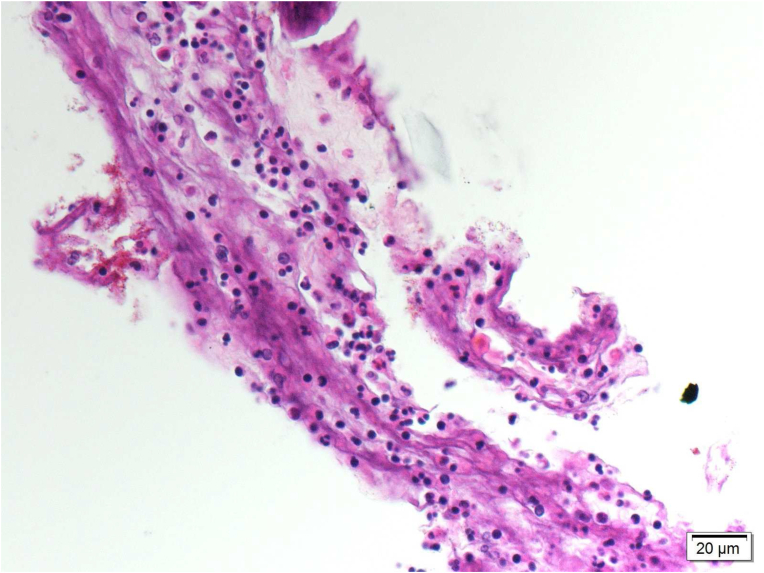
Hematoxylin and eosin stain (×400) showing a small piece of amniotic membrane stroma infiltrated with mixed acute and chronic inflammatory cells.

Histopathology indicated that a small piece of amniotic membrane stroma had been infiltrated with mixed types of acute and chronic inflammatory cells (Fig. 2). Gram staining indicated infiltration of mixed-type inflammatory cells and gram-positive cocci. Acid-fast bacilli and GMS strains were negative for other organisms. These findings indicated subacute inflammation with gram-positive cocci. Corneal scraping cultures indicated *Streptococcus mitis* and *Streptococcus oralis*. The antibiotic regimen was thus maintained. However, the patient developed a persistent epithelial defect with severe thinning that was unresponsive to topical management. Hence, we elected to perform a conjunctival flap because of the poor visual potential.

## 3 Discussion

In patients with bacterial keratitis, better outcomes have been reported with AMT than antibiotic therapy alone.^2^ In addition to its antibacterial effect, the AM has been reported to be effective at managing viral and fungal corneal ulcers.^3^^,^^4^

However, microbial keratitis after AM transplantation has been previously reported, at an incidence of 9.2%.^5^ Notably, the incidence decreased to 1.6% when donor tissue was prepared by a commercial laboratory using the Good Tissue Banking Practices set forth by the United States Federal Drug Administration compared to tissue preparation in a research laboratory.^6^

Al-Kharashi et al. reported that amniotic tissue prepared in a commercial laboratory or by appropriately qualified eye bank personnel may be used for AMT in eyes with persistent corneal epithelial defects with minimal risk of microbial keratitis in the first postoperative month. Their study evaluated the incidence of early onset (<30 days) and late-onset (>30 days) microbial keratitis after treatment of persistent corneal epithelial defects using tissue acquired from a commercial laboratory or prepared by our institutional eye bank. They observed no cases of early onset microbial keratitis in patients who received commercially acquired or locally prepared tissue. However, there were seven cases (9.7%) of late-onset microbial keratitis associated with the use of commercially prepared tissue compared to that associated with the use of locally prepared tissue. In all positive cultures, gram-positive organisms were most commonly isolated and mainly comprised Staphylococcus species.^5^

Alternatively, Marangon et al. reported that all cases of early onset infection post-AMT involved non-commercial laboratory-prepared tissue. They also found that gram-positive isolates were the most common post-AMT infections.^6^ Das et al. reported *Aspergillus* species isolated from the cornea, four weeks after AMT.^7^ In this case report, our patient received tissue that had been prepared under good tissue banking practices by a technician certified by the Eye Bank Association of America at the King Khaled Eye Specialist Hospital Eye Bank.

We presumed that our patient had three possible sources of infection: long-term use of bandage contact lens, compromised ocular surface due to uncontrolled diabetes mellitus, and application of topical steroids.

## 4 Conclusions

Despite the anti-inflammatory effect of AM, the infiltrate was localized to the basement membrane of the AM. The AM can act as a barrier against the infiltration of polymorphonuclear leukocytes from the tear film and microbial invasion into the cornea. Amniotic tissue prepared by appropriately qualified eye bank personnel may still carry a risk of infection; hence, prophylactic antibiotics are necessary.

## Patient consent

The study was approved by the Institutional Research Board (2025-CR) at King Khaled Eye Specialist Hospital. The tenets of the Declaration of Helsinki were followed at each step of the study.

## Funding

No funding or grant support was received.

## Availability of data and materials

All data generated or analyzed during this study are included in this published article.

## Authorship

All authors attest that they meet the current ICMJE criteria for authorship.

## Declaration of competing interest

The authors declare no potential conflicts of interest with respect to the research and/or publication of this article. The authors have no financial disclosures or potential conflicts of interest with respect to the research to disclose (SA, SA).
